# Perinatal asphyxia: CNS development and deficits with delayed onset

**DOI:** 10.3389/fnins.2014.00047

**Published:** 2014-03-26

**Authors:** Mario Herrera-Marschitz, Tanya Neira-Pena, Edgardo Rojas-Mancilla, Pablo Espina-Marchant, Daniela Esmar, Ronald Perez, Valentina Muñoz, Manuel Gutierrez-Hernandez, Benjamin Rivera, Nicola Simola, Diego Bustamante, Paola Morales, Peter J. Gebicke-Haerter

**Affiliations:** ^1^Millenium Institute BNI-ChileSantiago, Chile; ^2^Department of Molecular and Clinical Pharmacology, ICBM, Medical Faculty, University of ChileSantiago, Chile; ^3^Department of Chemical-Biological Science, University Bernardo O'HigginsSantiago, Chile; ^4^Department of Biomedical Sciences, Section of Neuropsychopharmacology, Cagliari UniversityCagliari, Italy; ^5^Department of Psychopharmacology, Central Institute of Mental Health J5Mannheim, Germany

**Keywords:** obstetric complications, neonatal hypoxic ischemic encephalopathy, sentinel proteins, poly(ADP-ribose) polymerase, plasticity, behavior, cognition, development

## Abstract

Perinatal asphyxia constitutes a prototype of obstetric complications occurring when pulmonary oxygenation is delayed or interrupted. The primary insult relates to the duration of the period lacking oxygenation, leading to death if not re-established. Re-oxygenation leads to a secondary insult, related to a cascade of biochemical events required for restoring proper function. Perinatal asphyxia interferes with neonatal development, resulting in long-term deficits associated to mental and neurological diseases with delayed clinical onset, by mechanisms not yet clarified. In the experimental scenario, the effects observed long after perinatal asphyxia have been explained by overexpression of sentinel proteins, such as poly(ADP-ribose) polymerase-1 (PARP-1), competing for NAD^+^ during re-oxygenation, leading to the idea that sentinel protein inhibition constitutes a suitable therapeutic strategy. Asphyxia induces transcriptional activation of pro-inflammatory factors, in tandem with PARP-1 overactivation, and pharmacologically induced PARP-1 inhibition also down-regulates the expression of proinflammatory cytokines. Nicotinamide has been proposed as a suitable PARP-1 inhibitor. Its effect has been studied in an experimental model of global hypoxia in rats. In that model, the insult is induced by immersing rat fetus into a water bath for various periods of time. Following asphyxia, the pups are delivered, treated, and nursed by surrogate dams, pending further experiments. Nicotinamide rapidly distributes into the brain following systemic administration, reaching steady state concentrations sufficient to inhibit PARP-1 activity for several hours, preventing several of the long-term consequences of perinatal asphyxia, supporting the idea that nicotinamide constitutes a lead for exploring compounds with similar or better pharmacological profiles.

## Introduction

It has been proposed that metabolic and environmental insults occurring during early development stages (i.e., pre- and perinatal) lead to psychiatric manifestations with an onset at adolescence and/or young adulthood stages (Basovich, 2010). Accordingly, epidemiologic studies have shown that pre- and perinatal factors, like maternal infections (Brown and Derkits, 2010) or hypoxia are related to schizophrenia (Cannon et al., 2002, 2008). Furthermore, high maternal anxiety during pregnancy has been associated to attention deficit and hyperactivity disorders, together with cognitive impairments and emotional symptoms (Van den Bergh et al., 2005, 2006; Loomans et al., 2011, 2012). In animal models, different risk factors have been identified occurring early in life, including maternal stress during pregnancy (Baier et al., 2012), early postnatal maternal separation (Zhu et al., 2010; Hulshof et al., 2011), neonatal bedding stress (Green et al., 2011), infections (Landreau et al., 2012) and asphyctic injury (Dell'Anna et al., 1991; Morales et al., 2011), promoting cognitive deficits, anxiety, and other behavioral disturbances at adulthood. Obstetric complications, in particular, are associated to psychiatric and neurological disorders (Cannon et al., 2002), hypoxia being a recurrent co-factor, adversely priming brain development by mechanisms not yet established (Low, 2004; Basovich, 2010; Herrera-Marschitz et al., 2011).

Delay in starting pulmonary ventilation at birth implies decrease of oxygen saturation in blood and reduced oxygen supply to the brain, which depends on aerobic metabolism for maintaining the respiratory chain and mitochondrial ATPase activity. Whenever hypoxia is sustained, there is a switch to glycolysis, which for neurons is a poor metabolic alternative, because of low stores of glucose in brain tissue and deficient ATP output by the glycolysis pathway. Glycolysis implies production of lactate, which is accumulated in extracellular compartments, causing acidosis, although it has also been suggested that lactate is an energy source for neurons (see Wyss et al., 2011). Prolonged hypoxia impairs gene expression, decreases transcription and translation, as well as activation of genes, such as hypoxia inducible factor (HIF) and its target molecules (Iyer et al., 1998).

Re-oxygenation is a requisite for survival, but it encompasses uneven metabolism, with metabolically privileged (e.g., heart, brain, and adrenal medulla) and less privileged (e.g., muscles, kidneys, and carcass) organs, including uneven distribution in brain regions. During the re-oxygenation period extracellular glutamate levels are increased, enhancing the activation of Na^+^/K^+^ ATPase, increasing further ATP consumption. Extracellular glutamate levels override the buffer capacity of astrocytes, resulting in sustained overactivation of glutamate receptors, mainly of the N-methyl-D-aspartate (NMDA) subtype, increasing Ca^2+^ conductance and further improper homeostasis. The metabolic crisis implies lactate accumulation and acidosis, differently reflected along development and brain regions. Indeed, we found that lactate levels remained increased (>2-fold) in neostriatum, but not in other regions of the basal ganglia of severely asphyxia-exposed, compared to control rats assayed 8 days after birth (Chen et al., 1997a).

**Figure 1:**
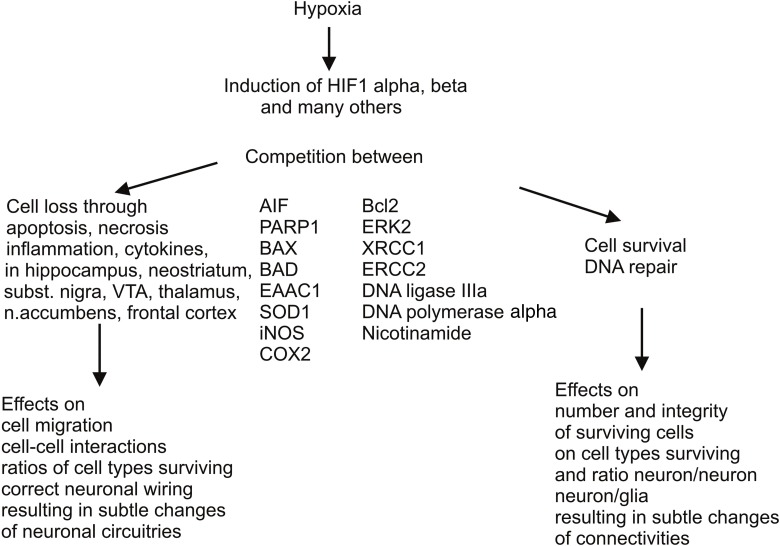
**Antagonistic molecular and cell cascades elicited by perinatal asphyxia**. There are two important molecular and cell cascades elicited by perinatal hypoxic insults, one leading to removal of cells damaged by the reduced supply of oxygen, implying activation of ubiquitination, peroxisomal, and caspase pathways, resulting in apoptosis or necrosis, the latter encompassing mild to severe inflammation. The other cascade is activated for compensating cell loss by multiple mechanisms of DNA repair. Naturally occurring nicotinamide with its various mechanisms of action could support these compensatory mechanisms if given systemically as a therapeutic means. In any case, both cell loss and cell rescue eventually entail more or less subtle consequences on brain development, neuronal wiring, and neuron glia interactions. These tentatively negative consequences are probably reinforced by additional negative impact during development (puberty and adolescence), resulting in psychiatric disorders if not aggravating neurological deficits.

All these changes are inherent biological reactions that result in partial recovery, but also in sustained overexpression of alternative or even antagonistic metabolic pathways, and molecular and cell cascades, prolonging the energy deficit and oxidative stress, associated with further cell damage and apoptotic or necrotic cell death (Figure 1). Molecular and cell cascades involved in removal of cells damaged by the reduced supply of oxygen are the ubiquitination, peroxisomal, and caspase pathways, whereas optional, compensatory cascades elicit multiple mechanisms of DNA repair, for prevention of cell loss or salvage of cells, respectively.

Oxidative stress is inherent to re-oxygenation, resulting in overactivation, but also inactivation of a number of buffering enzymes; including those modulating the activity of mitochondria (see Gitto et al., 2002). In the clinical scenario, resuscitation may even imply hyperoxemia, leading to further production of free radicals and oxidative stress, worsening brain injury (Davis et al., 2004; Solberg et al., 2007; see Kapadia et al., 2013). Therefore, hypothermia has been proposed as a therapeutic intervention, targeting functional hyperoxemia and oxidative stress, decreasing the metabolic demands, attenuating the disturbances of metabolism elicited by re-oxygenation, preventing the short- and long-term consequences of perinatal asphyxia (Gunn et al., 1998; Engidawork et al., 2001). Indeed, hypothermia is now recommended as a routine intervention in the clinical practice (Azzopardi et al., 2009; Hagmann et al., 2011), although there is still concern about a narrow therapeutic window (Roelfsema et al., 2004; see also Van den Broek et al., 2010), and doubts about whether the treatment can really prevent the long-term consequences of perinatal asphyxia.

## Cell death and regional vulnerability: endpoints for the outcome of perinatal asphyxia

Delayed cell death is an important end-point of perinatal asphyxia, associated to caspase-dependent and caspase-independent mechanisms (Northington et al., 2001). Several pro-apoptotic proteins are increased following perinatal asphyxia, including B-cell lymphoma-2 (Bcl-2) associated X (BAX), and Bcl-2 associated death (BAD) factors, but also anti-apoptotic proteins, including Bcl-2, extracellular signal-regulated kinase 2 (ERK2), and basic fibroblast growth factor (bFGF) (Morales et al., 2008). Extensive and regionally selective nuclear fragmentation has been observed in control and asphyxia-exposed rat pups, depending upon the stage of development and the analyzed brain region (Dell'Anna et al., 1997). Consequently, signs of apoptosis have been found in normal and asphyxia-exposed animals, independently upon the severity of the insult. However, in neocortex, neostriatum and mesencephalon a significant increase of apoptotic cells is only observed in asphyxia-exposed animals, which documents regional vulnerability (Dell'Anna et al., 1997; Neira-Peña et al., 2014).

This regional vulnerability of the basal ganglia to anoxia/ischemia has been reported by several groups (Pasternak et al., 1991; Pastuzko, 1994; Cowan et al., 2003; Miller et al., 2005; Barkovich, 2006; Ferrari et al., 2011). It can be explained either by (1) the severity of the insult; (2) the local metabolic imbalance during the re-oxygenation period, and/or (3) the developmental stage of the affected regions. Immaturity of a particular brain region plays a role, because the insult affects the initial plastic changes required for establishing neurocircuitries and synaptogenesis. Furthermore, the re-establishment of homeostasis consumes extra energy that competes with the demands required for consolidating neurocircuitries and synapsis consolidation.

Development of neurocircuitries is based on a strict sequential programming. For instance, in terms of dopamine pathways, it is well-established that at P1 the brain of rats possesses a similar number of dopamine cell bodies as in adulthood, but, although dopamine fibers start to invade the neostriatum before birth (Seiger and Olson, 1973), dopamine-containing axon terminals reach a peak at the fourth postnatal week, and a mature targeting is only achieved after several postnatal weeks, when patches are replaced by a diffuse innervation pattern (Olson and Seiger, 1972; Seiger and Olson, 1973; Voorn et al., 1988; Antonopoulos et al., 2002; Loizou, 1972). From this time on, dopaminergic axons continue to grow at a slow rate during adulthood (Loizou, 1972; Voorn et al., 1988), followed by naturally occurring waves of dopamine cell death (Oo and Burke, 1997; Antonopoulos et al., 2002). The neocortex and hippocampus are also immature at postnatal stages. In the rat, neocortical pyramidal projections become physiologically viable only 1 week after birth (Li and Martin, 2000; Meng and Martin, 2003; Meng et al., 2004), and in humans the prefrontal cortex achieves a full mature stage long after postadolescent stages (Sowell et al., 1999; Segalowitz and Davies, 2004).

Henceforth, it is not surprising that mesostriatal, mesolimbic and mesocortical monoamine pathways are vulnerable to perinatal asphyxia. Long-lasting decreases of tyrosine hydroxylase (TH)-positive cell bodies have been observed in substantia nigra and ventral tegmental area (VTA) of rats exposed to severe perinatal asphyxia (Chen et al., 1995, 1997b), together with decreased dopamine utilization in neostriatum, accumbens, and olfactory tubercle (Ungethüm et al., 1996; Bustamante et al., 2003) 1 month after birth. A decrease in D-amphetamine stimulated dopamine release was also observed when asphyxia-exposed animals were evaluated using *in vivo* microdialysis 3 months after birth (Bustamante et al., 2007). In agreement, decreases in TH immunohistochemistry have been observed in neostriatum, hippocampus, thalamus, frontal cortex, and cerebellum of asphyxia-exposed rats evaluated 1–3 months after birth (Kohlhauser et al., 1999a,b). However, in the same animals, the excitatory amino acid carrier 1 (EAAC1) was increased in the frontal cortex (Kohlhauser et al., 1999b). The regional selectivity of the insult has been further investigated with triple organotypic cultures, finding a selective decrease in the number of dopamine neurons in cultures from asphyxia-exposed animals. In contrast, in the same cultures, nitric oxide synthase (NOS) positive neurons were increased in substantia nigra, decreased in neostriatum, and not changed in neocortex, again underlining the regionally different vulnerability (Klawitter et al., 2007). Neurite length and branching of neurons with dopamine and NOS phenotypes were also decreased in rats exposed to perinatal asphyxia (Morales et al., 2003; Klawitter et al., 2005, 2007). A similar effect has been observed in hippocampus. Neurite length and branching, as well as the expression of synaptophysin and postsynaptic density protein 95 (PSD95), pre- and postsynaptic markers, respectively, were found to be decreased at P30 in tissue from asphyxia-exposed animals (Rojas-Mancilla et al., 2013).

## Sentinel proteins

Suppression and/or overactivation of gene expression occur immediately or during the re-oxygenation period following perinatal asphyxia (Labudova et al., 1999; Mosgoeller et al., 2000; Seidl et al., 2000; Lubec et al., 2002). When DNA integrity is compromised, a number of sentinel proteins is activated, including poly(ADP-ribose) polymerases (PARPs) (Amé et al., 2004); X-Ray Cross Complementing Factor 1 (XRCC1) (Green et al., 1992); DNA ligase IIIα (Leppard et al., 2003); DNA polymerase β (Wilson, 1998; Mishra et al., 2003); Excision Repair Cross-Complementing Rodent Repair Group 2 (ERCC2) (Sung et al., 1993; Chiappe-Gutierrez et al., 1998; Lubec et al., 2002), and DNA-dependent protein kinases (De Murcia and Menissier de Murcia, 1994).

PARP proteins transfer adenosine diphosphate (ADP)-riboses from nicotinamide dinucleotide (NAD^+^) to glutamic and aspartic residues of the PARPs and their substrates. PARPs also catalyze the polymerization of ADP-riboses via glycosidic bonds, creating long and branched ADP-ribose polymers. PARP-1 is the most abundant and conserved member of a large superfamily comprising at least 18 PARP proteins, encoded by different genes, but displaying a conserved catalytic domain. PARP-1 is involved in DNA repair, but it also promotes cell death (see De Murcia and Menissier de Murcia, 1994; Kauppinen and Swanson, 2007; Cohen-Armon, 2008). When DNA damage is mild, PARP-1 is involved in the maintenance of chromatin integrity, by signaling cell-cycle arrest or activating DNA repairing molecular cascades. Furthermore, PARP-1 is involved in the regulation of cell proliferation and differentiation, modulating the transcription of several inflammatory signals, including nuclear factor κB (NF-κB) (Hassa and Hottinger, 1999). Excessive PARP-1 activation leads to NAD^+^ exhaustion and energy crisis (Berger, 1985), and to caspase-independent apoptosis, via translocation of the mitochondrial pro-apoptotic protein Apoptosis-Inducing Factor (AIF) to the nucleus, producing nuclear condensation (Jiang et al., 1996; Yu et al., 2002; Hong et al., 2004). PARP-1 has been involved in the long-term effects produced by perinatal asphyxia (Martin et al., 2005), interacting with XRCC1, DNA ligase IIIα, and DNA polymerase-β, working in tandem to repair single-strand breaks. DNA ligase IIIα has a N-terminal zinc finger interacting with the DNA binding domain of PARP-1 and DNA strand breaks. Further, DNA Ligase IIIα interacts with XRCC1, forming a DNA Ligase IIIα-XRCC1 complex (see Ellenberger and Tomkinson, 2008; Odell et al., 2011; Simsek et al., 2011), and DNA polymerase-β can couple to PARP-1, DNA Ligase IIIα, and XRCC1, providing the overall stability of the repair complex, promoting catalysis and fidelity (Sawaya et al., 1997). It has been shown that PARP-1, DNA polymerase-β, and XRCC1 expression are increased by hypoxia in newborn piglets (Mishra et al., 2003) and rats (Chiappe-Gutierrez et al., 1998). With an ischemic preconditioning model, Li et al. (2007) demonstrated a 5-fold increase of XRCC1 levels 30 min after ischemia, reaching a maximal expression after 4 h. DNA polymerase-β and DNA ligase IIIα levels were also increased, and co-expressed in neuron and glial cells. PARP-1 can be phosphorylated by ERK, probably via the isoform 2 (ERK2), as a requirement for maximal PARP-1 activation after DNA damage (Kauppinen et al., 2006). Nevertheless, Cohen-Armon et al. (2007) reported evidence for PARP-1 activation by phosphorylated ERK2, in the absence of DNA damage, via the signaling cascade of the transcription factor ETS domain-containing protein 1 (Elk1), increasing the expression of the immediate early gene *c-fos*, stimulating cell growth and differentiation. Thus, cell type specificity and regional distribution of sentinel proteins may provide regulatory mechanisms by which the long-term effects of metabolic insults occurring at birth are heterogeneous, targeting some, but leaving other brain regions apparently untouched.

## PARP-1 activity and inflammatory signaling

In the developing brain, re-oxygenation leads to oxidative stress, generating reactive oxygen species (ROS) and H_2_O_2_ accumulation, a dangerous outcome, because the immature brain expresses low activity of glutathione peroxidase (GPx) and catalase, the enzymes ameliorating the damaging effects of superoxide and hydrogen peroxide, respectively (Lafemina et al., 2006). While superoxide dismutase (SOD) is the only known enzymatic scavenger of extracellular superoxide levels, its overexpression in the cytosol (SOD1) of neonatal animals increases hypoxic-ischemic brain injury (Ditelberg et al., 1996), together with H_2_O_2_ accumulation (Lafemina et al., 2006). The metabolic insult enhances nitric oxide (NO) synthesis via inducible nitric oxide synthase (iNOS), producing large amounts of NO over extended periods, damaging cell constituents, promoting peroxynitrite formation, and lipid and protein peroxidation (Bonfocco et al., 1995), as well as activation of cyclooxygenase-2 (COX-2) (Nogawa et al., 1998), eliciting inflammatory cascades (see Hagberg et al., 1996).

DNA damage elicited by perinatal asphyxia implies PARP-1 overactivation, promoting NF-κB translocation and expression of proinflammatory cytokines (Ullrich et al., 2001), a phenomenon associated with microglial migration toward the site of neuronal injury (see Skaper, 2003; Gagne et al., 2008). Conversely, PARP-1 inhibition suppresses NF-κB dependent gene transcription in microglia (Kauppinen et al., 2008). In a model of bilateral carotid occlusion-reperfusion in rats, Kauppinen et al. (2009) demonstrated that treatment with the selective PARP-1 inhibitor PJ34 ([*N*-(6-Oxo-5, 6-dihydrophenanthridin-2-yl)-*N*, *N*-dimethylacetamide-HCl]), 48 h later, suppressed ischemia-induced microglial activation, enhancing long-term neuronal survival, neurogenesis, and spatial memory. Also, in a model of forebrain ischemia, PJ34 produced a near-complete inhibition of microglia activation and a significant reduction of neuronal death in the hippocampus (Hamby et al., 2007). Furthermore, it was reported that minocycline, a tetracycline derivative with anti-inflammatory properties (see Carty et al., 2008), protected neuronal cultures against genotoxic agents, such as N-methyl-N-nitro-N-nitrosoguanidine (MNNG) and 1, 3-morpholinosydonimine (SIN-1). Interestingly, minocycline also inhibited PARP-1 activity (Alano et al., 2006). It has been suggested that perinatal asphyxia induces transcriptional activation of pro-inflammatory factors, in tandem with PARP-1 overactivation (Neira-Peña et al., 2013). Indeed, there is an increase of mRNA levels of IL-1β and TNF-α in mesencephalon and hippocampus of asphyxia-exposed animals, in agreement with reports showing that TNF-α, IL-1β, as well as IL-6 modulate cell death, vascular permeability and recruitment of peripheral blood cells into the CNS (see Deverman and Patterson, 2009; Russo et al., 2011), supporting the idea that inflammasomes, a large intracellular multiprotein complex (see Schroder and Tschopp, 2010), plays a central role in the activation of IL-1β, via NACHT, LRR, and PYD domain-containing protein 3 (NALP3). NLRP3 recruits caspase-1, which cleaves pro-IL1β, generating the active form of IL-1β. Inflammasomes can also be activated by damage-associated molecular pattern molecules (DAMPs), known as danger-associated molecular pattern molecules, including extracellular ATP and molecules related to metabolic stress (see Schroder and Tschopp, 2010; Fann et al., 2013).

A relationship between serum levels of IL-1β, IL-6, and TNF-α and the outcome of infants that have suffered of perinatal asphyxia has been observed in humans (Foster-Barber et al., 2001), suggesting that proinflammatory cytokines have a predictive value. Indeed, a study of blood and CSF obtained during the first 24 h of life from infants who suffered hypoxic ischemic encephalopathy showed an increase in IL-1β, IL-6, and TNF-α levels, compared to control infants (Aly et al., 2006).

## Targets for neuroprotection

It is proposed that PARP-1 inhibition is a target for neuroprotection following hypoxia/ischemia. Several PARP inhibitors, with increasing degrees of potency, have been shown to decrease brain damage, improving the neurological outcome of perinatal brain injury (Zhang et al., 1995; Ducrocq et al., 2000; Sakakibara et al., 2000; see Virag and Szabo, 2002; Jagtap and Szabo, 2005; Kauppinen et al., 2009).

The idea that PARP-1 activation is beneficial has also been explored, depending upon the actual levels of cellular NAD^+^. While PARP inhibitors offer remarkable protection under conditions of NAD^+^ and ATP depletion, inhibition of PARP-1 in the presence of NAD^+^ sensitizes cells to DNA damage, and subsequent increase of cell death (Nagayama et al., 2000). Also, inhibition of PARP-1 induces apoptosis in rapidly dividing cells (Saldeen and Welsh, 1998), probably by blocking the access of repairing enzymes. Thus, PARP-1 acts as both a cell survival and cell death-inducing factor by regulation of DNA repair, chromatin remodeling, and regulation of transcription.

Nicotinamide is a reference compound regarding PARP-1 inhibition (Virag and Szabo, 2002). Because of its relative low potency, it is proposed to be useful when treating young animals, antagonizing the effects elicited by PARP-1 overactivation without impairing DNA repair or cell proliferation. Nicotinamide has also been proposed as an antioxidant compound (Wan et al., 1999; Yan et al., 1999), and its pharmacodynamics can provide advantages over more selective compounds (Griffin et al., 2013; Shetty et al., 2013; Turunc Bayrakdar et al., 2014). Apparently, it also helps to replenish the reduced pool of NAD^+^ by being metabolized through nicotinamide phosphoribosyltransferase; (NAMPT) (Pittelli et al., 2014).

## An experimental working model for perinatal asphyxia

While the short-and long-term clinical outcomes of perinatal asphyxia are well-established, pre-clinical research is still at an exploratory phase, mainly because of a lack of consensus on a reliable and predictable experimental model. A model for investigating the issue was proposed at the Karolinska Institutet, Stockholm, Sweden, at the beginning of the nineties (Bjelke et al., 1991; Andersson et al., 1992; Herrera-Marschitz et al., 1993). The model starts by evaluating the oestral cycle of young female rats for a programmed mating. A vaginal frottis is taken for evaluating the cycle, and the female is exposed to a male at the time of the pro-oestrous for 1 night. Thereafter the presence of a vaginal clot is evaluated, to predict the exact time of delivery (22 days after a vaginal clot has been recorded). At the time of delivery, a first spontaneous delivery can be observed before the dams are anaesthetized, neck dislocated, and subjected to a caesarean section and hysterectomy. The uterine horns containing the fetus are immediately immersed into a water bath at 37°C for various periods of time (0–22 min). Following asphyxia, the pups are removed from the uterine horns and resuscitated. Additional efforts and care are taken to induce and maintain pulmonary breathing. Several laboratories are running the model, although it has criticized that it works with on term pups and not with neonates at P7, arguing that the brain of neonate rats is premature when compared to the neonatal human brain, a statement mainly referring to the neocortex (see Romijn et al., 1991), and the pattern of oligodendrocyte lineage progression required for cerebral myelination (Craig et al., 2003). Our view is that the degree of maturity depends upon the tissue and functions selected for the comparisons, vulnerability relating to both the timing and the location of the insult (Craig et al., 2003, see also De Louw et al., 2002). In agreement, it has been reported that susceptibility to hypoxia-ischemia induced by common carotid artery ligation and hypoxemia is greater when performed at P7 rather than at earlier postnatal periods (Towfighi et al., 1997).

Our approach is rather pragmatic, inducing asphyxia at the time when rats are delivered. Thus, our model: (1) mimics well-some relevant aspects of human delivery; (2) it is largely non-invasive; (3) it allows studying short- and long-term consequences of the insult in the same preparation, and (4) it is highly reproducible among laboratories. Gert Lubec and co-workers in Vienna (Austria) (Lubec et al., 1997a,b; Seidl et al., 2000) have stressed the issue that the model is suitable for studying the early phase of perinatal asphyxia, as observed in the clinical setup, performed during delivery, acknowledging the fact that the model implies oxygen interruption, but not any additional lesions, including, by example, vessel occlusion. Moreover, it is followed by hypoxemia, acidosis and hypercapnia, mandatory criteria for a clinically relevant model of perinatal asphyxia (Seidl et al., 2000).

Pups exposed to zero or mild asphyxia start breathing with a gluttonous gasp, which is rapidly replaced by regular and synchronized breathing. For pups exposed to longer periods of asphyxia (19–21 min), resuscitation implies expert and skillful handling, taking a long time (4–6 min) for a first gasping, and even longer time for establishing a more or less regular breathing, always supported by gasping. Surrogate dams can nurture the pups, whether long-term experiments are desirable.

With that rat model (Bjelke et al., 1991; Andersson et al., 1992; Herrera-Marschitz et al., 1993), we reported that nicotinamide prevents several of the neuronal (Klawitter et al., 2006, 2007; Morales et al., 2010), neurochemical (Bustamante et al., 2003, 2007) and behavioral (Simola et al., 2008; Morales et al., 2010) effects produced by perinatal asphyxia. With microdialysis experiments performed 1 h after birth, it was found that after a single systemic dose (0.8 mmol/kg, i.p.), nicotinamide rapidly distributed along different compartments including the brain, achieving concentrations larger than 30 μ M for longer than 30 min, producing a long-lasting inhibition of PARP-1 activity in mesencephalon, telencephalon, and heart of asphyxia-exposed and control animals (Allende-Castro et al., 2012). Interestingly, PARP-1 inhibitors, which do not reach the brain at enough sustained concentrations, such as 1,3-dimethylxanthine (theophylline) (Geraets et al., 2006; Allende-Castro et al., 2012), do not protect against the long-term effects elicited by perinatal asphyxia, although it has been shown that theophylline reduces inflammation and innate immunity (Peters-Golden et al., 2005).

## Cognitive deficits

Motor and cognitive alterations of variable severity, including cerebral palsy, seizures, spasticity, attention deficit, hyperactivity, mental retardation, and other neuropsychiatric syndromes with delayed clinical onset have been associated to perinatal asphyxia (du Plessis and Volpe, 2002; Van Erp et al., 2002; Kaufman et al., 2003; Vannuci and Hagberg, 2004; De Hann et al., 2006; Odd et al., 2009). In the rat, several studies have investigated the behavioral effects associated with perinatal asphyxia, addressing motor function (Bjelke et al., 1991; Chen et al., 1995), emotional behavior (Dell'Anna et al., 1991; Hoeger et al., 2000; Venerosi et al., 2004, 2006; Morales et al., 2010), and spatial memory (Boksa et al., 1995; Iuvone et al., 1996; Hoeger et al., 2000, 2006; Loidl et al., 2000; Van de Berg et al., 2003; Venerosi et al., 2004).

We have investigated whether perinatal asphyxia produces long-term effects on cognitive performance, using an object recognition test (Ennaceur and Delacour, 1988), based on the ability of the rat to discriminate between objects differing in shape and color, without any association to rewarding or aversive stimuli. During a first session, two copies of the same object are presented to the rat for a short period, and then again, during a second session, when one of the previously presented copies is replaced by a novel object, similar in size, but different in shape and/or color. The idea is that the rat has to recognize the novel object, spending longer time exploring the novel than the previously presented object. The first and the second sessions can be separated by different time intervals, for evaluating learning consolidation. A good memory would be able to recognize a previously presented object after a long elapsing time between a first and a second session, implying that the animal concentrates on exploring the novel object. In our studies, we used 15 or 60 min intervals, and the animals were studied at 3 months of age (Simola et al., 2008). No differences were observed between animals exposed to severe asphyxia and control animals when a 15 min interval elapsed between the first and the second session. However, when 60 min elapsed between the first and second session, asphyxia-exposed animals spent less time exploring the novel object, indicating that asphyxia-exposed rats could not recognize its novelty.

Notably, the total exploration time was not affected by the insult, suggesting that asphyctic rats do not have non-specific deficits in object discrimination, but rather display a specific deficit in non-spatial working memory (Simola et al., 2008). This is a straightforward experiment showing a subtle consequence of a metabolic insult (hypoxia) occurring at birth, impairing a cognitive function that shows up only after a proper challenge. Results in line with these have also been obtained by subsequent studies in either rats or mice subjected to the same experimental model of perinatal asphyxia (Hutton et al., 2009; Strackx et al., 2010). This impairment in object recognition is very much reminiscent of the clinical experience revealing effects only when the child starts primary school (see Odd et al., 2009; Strackx et al., 2010).

Nicotinamide prevented the effect of perinatal asphyxia on novel object recognition evaluated after a 60 min elapse. Nicotinamide did not affect the pattern observed in the corresponding control rats (Morales et al., 2010). The effect of nicotinamide on novel object recognition deficit induced by perinatal asphyxia was related to that on hippocampus delayed cell death, suggesting an effect on a specific neuroanatomical substrate.

## Conclusions

Perinatal asphyxia is still a health concern worldwide, a risk factor for several mental and neurological disorders showing a delayed clinical onset.

**Figure 2:**
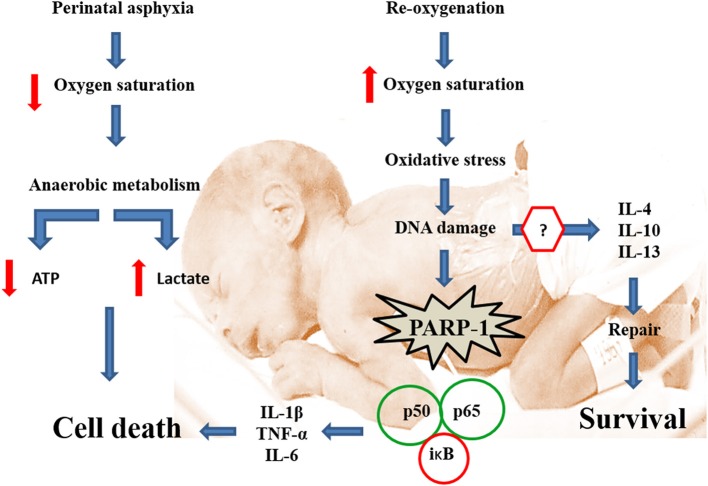
**Transcriptional activation of pro-inflammatory signaling in tandem with PARP-1 overactivation**. Perinatal asphyxia decreases oxygen saturation in blood, leading to a switch from aerobic to a less efficient anaerobic metabolism involving lactate accumulation, acidosis and cell death. Re-oxygenation is essential for survival. Nevertheless, re-oxygenation induces oxidative stress, damaging several biomolecules including DNA. In response to DNA damage, PARP-1 increases its activity, recruiting the DNA repair machine. PARP-1 overactivation modifies several target proteins via poly (ADP-ribose) polymers (pADPr). One of the targets of pADPr ribosylation is NF-κB, whose p65 subunit is translocated to the nucleus, activating the transcription of pro-inflammatory (e.g., TNF-α, IL-1β, and IL-6), but also anti-inflammatory (e.g., IL-4, IL-10; IL-13) cytokines. The balance between pro- and anti-inflammatory cytokines is crucial for determining cell survival or death.

As shown by Figure 2, hypoxia implies a severe energetic crisis, leading to death if re-oxygenation is not promptly restored. Decrease of oxygen saturation implies a shift to anaerobic metabolism for preserving the fundamental demands of energy. Anaerobic metabolism is, however, inefficient for providing the energy required by a sophisticated, high consuming, but low energy-storing organ, such as the brain. A decrease of energy implies low metabolism, which can be enough for maintaining basic functioning, competing, however, with the high metabolism required for development of neuronal networks and neurocircuitries. Anaerobic metabolism implies also lactate accumulation and acidosis. Whether oxygen supply is not restored, the reserves are exhausted. Energy can still be compartmentalized among privileged and less privileged brain regions, without consideration, however, about the implications for long-term development. Re-oxygenation is a requisite for survival, but the kinetics of oxygen supply implies global and/or local hyperoxemia, free radicals accumulation, oxidative stress, cell damage, and overactivation of defense mechanisms, including sentinel proteins, but also inflammatory cascades and further cell stress.

Thus, the functional constraints produced by the lack of oxygen are exacerbated by and during the re-oxygenation period, implying oxidative stress, synthesis, and release of metabolic by-products delaying the onset of proper homeostasis and recovery. A number of sentinel proteins are rapidly activated whenever there is a risk of genome damage, stimulating base excision repair. PARP-1 plays a pivotal role for repairing damaged DNA, eliciting also caspase-independent cell death when repair is not viable. PARP-1 overactivation leads to NAD^+^ exhaustion, worsening the energy crisis, which is the basis for the hypothesis that PARP-1 is a suitable target for therapeutic interventions preventing the long-term effects of perinatal asphyxia, nicotinamide being a prototype for counteracting PARP-1 overactivation. Asphyxia also induces transcriptional activation of pro-inflammatory factors, in tandem with PARP-1 overactivation. In agreement, pharmacologically induced PARP-1 inhibition also down-regulates the expression of proinflammatory cytokines.

### Conflict of interest statement

The authors declare that the research was conducted in the absence of any commercial or financial relationships that could be construed as a potential conflict of interest.

## Abbreviations

ADP: adenosine diphosphate
AIF: apoptosis-inducing factor
ATP: adenosine triphosphate
ATPase: ATP polymerase
BAD: Bcl-2-associated death factor
BAX: Bcl-2-associated X protein
BBB: blood-brain barrier
bFGF: basic fibroblast growth factor
Bcl-2: B-cell lymphoma-2
CA: *cornus Ammonis* (CA1, C2, C3)
CAM: cell adhesion molecule
CNS: central nervous system
COX-2: cyclooxygenase-2
CS: caesarean-delivered saline treated animal
CSF: cerebral spinal fluid
DAMPs: damage-associated molecular pattern molecules
DG: dentate gyrus of the hippocampus
DNA: deoxyribonucleic acid
EAAC1: excitatory amino acid carrier 1
Elk1: ETS domain-containing protein 1
ERCC2: excision repair cross-complementing rodent repair group 2
ERK: extracellular signal-regulated kinases
FGFR: bFGF receptors
FLRT3: leucine-rich repeat transmembrane protein
GPx: glutathione peroxidase
HIF: hypoxia inducible factor
IκB: inhibitor of kappa B protein
iNOS: inducible NOS
IL-1β/-6: interleukin-1β/-6
ICAM-1: TNFα adhesion molecule-1
L1: L1 CAM
LPS: lipopolysaccharide
MAP-2: microtubule-associated protein-2
MAPK: mitogen-activated protein kinase
MNNG: N-methyl-N-nitro-N-nitrosoguanidine
NACHT: LRR and PYD domains-containing protein 3 (cryopyrin)
NAD^+^: oxidized nicotinamide adenine dinucleotide
NADH: reduced nicotinamide adenine dinucleotide
NALP3: NACHT, LRR, and PYD domain-containing protein 3 (cryopyrin)
NAMPT: nicotinamide phosphoribosyltransferase
NF-κB: nuclear factor κB
NMDA: N-methyl-D-aspartate
NO: nitric oxide
NOS: nitric oxide synthase
nNOS: neuronal NOS
p65/p50: protein subunits of 65/50 k Dalton MW
P1: postnatal day 1
PARP-1: poly(ADP-ribose) polymerase-1
PIP2: phosphatidylinositol-4,5-bisphosphate
PJ34: [*N*-(6-Oxo-5, 6-dihydrophenanthridin-2-yl)-*N*, *N*-dimethylacetamide.HCl]
PKC: protein kinase C
pADPr: poly (ADP-ribose) polymers
PSD95: postsynaptic density protein 95
RhoA: ras homolog gene family, member A, small GTPase protein
ROS: reactive oxygen species
Sef: similar expression fgf gene
SEM: standard error of the means
SIN: 1, 3-morpholinosydonimine
SIRT: sirtuin
SOD: superoxide dismutase
Spry: sprouty
SRY: sex determining region Y
SVZ: subventricular zone
TH: tyrosine hydroxylase
Thy1: thymocyte differentiation antigen 1
TNF-α: tumor necrosis factor-alpha cytokine
TUNEL: TdT-mediated dUTP nick-end labeling
VTA: ventral tegmental area
XRCC1: X-ray cross complementing F factor 1.
